# Identification of the YABBY Gene Family in *Cerasus humilis* and Analysis of Expression Patterns During Different Growth Stages

**DOI:** 10.3390/biology14111511

**Published:** 2025-10-28

**Authors:** Weichao Ren, Shan Jiang, Lingyang Kong, Chenzhuo Yue, Lengleng Ma, Junbai Ma, Wei Ma, Xiubo Liu

**Affiliations:** 1 College of Pharmacy, Heilongjiang University of Chinese Medicine, Harbin 150040, China; 2 College of Jiamusi, Heilongjiang University of Chinese Medicine, Jiamusi 154007, China

**Keywords:** gene family, *Cerasus humilis*, growth and development, transcription factor, gene expression

## Abstract

**Simple Summary:**

We identified and analyzed the *Cerasus humilis YABBY* (*ChYABBY*) gene family and further explored the expression changes in *ChYABBYs* in different growth stages. At the same time, the changes in physiological indexes in different periods of fruit were measured, the correlation between them and *YABBY* gene expression was analyzed, and the possible regulatory mechanism was speculated. This research provides an important basis for further understanding the structure and function of the *ChYABBY* gene, and lays a foundation for the identification of YABBY genes in Rosaceae plants.

**Abstract:**

YABBY belongs to the family of plant-specific transcription factors, known for their role in plant morphology, growth, and development. Its name is derived from the first discovered member—the *YABBY1* gene of *Arabidopsis thaliana* (named due to its mutated phenotype showing a “Y-shaped” bifurcation). Despite extensive research across various plant species, no studies have conducted a genome-wide investigation of the YABBY gene family in *Cerasus humilis*. This study identified six *ChYABBY* (*Cerasus humilis YABBY*) genes distributed across five chromosomes through a comprehensive bioinformatic analysis of the *C. humilis* genome. The gene expression during the four growth phases was confirmed using real-time-quantitative fluorescent PCR (qPCR). *ChYABBY* is segmented into five distinct subfamilies. Genetic lineage analysis determined the close genetic relationship between the *YABBY* genes of *C. humilis* and *Malus pumila*. An examination of the gene architecture and preserved motifs revealed that *ChYABBY* typically comprises 5–6 introns, with motif1, motif2, and motif3 being preserved domains across all ChYABBY protein sequences. Promoter analysis suggests that *ChYABBY* genes play various roles in the growth and maturation of *C. humilis*. An examination of the homology revealed the absence of tandem replication in the ChYABBY gene family, with a single pair of fragment-replicating genes. The heat map and q-PCR results indicate that the expression of the *ChYABBY* gene is tissue-specific and correlates with some aspects of the fruit growth and development. This suggests a potential role for this gene family in fruit maturation. The determination of total sugar and total flavonoid content indicated that the content of the two substances was high when the fruit was green. The antioxidant capacity of the fruit at each stage was different. This research provides an important basis for further understanding the structure and function of the *ChYABBY* gene, and lays a foundation for the identification of *YABBY* genes in Rosaceae plants.

## 1. Introduction

Transcription factors play a vital role in regulating plant growth, development, and stress responses [1]. The YABBY gene family is a type of transcription factor specific to plants. The YABBY family, part of the zinc finger protein superfamily, is important in developing collateral organs during the growth and development of plants [2,3,4,5]. Two common domains exist within the YABBY family, namely, the N-terminal C2C2 domain, which facilitates protein interactions, and the C-terminal YABBY domain, responsible for protein-DNA interactions, both of which are extensively preserved across various species [6]. The YABBY transcription factor is crucial in regulating dorsal and abdominal polarity in plant tissues, the growth of leaves, development of plants, creation of flowers, growth of fruits, biosynthesis of secondary metabolism, and biological and non-biological stress processes in plants [5].

Six *YABBY* genes have been identified in *A. thaliana*, namely, *FILAMENTOUS FLOWER* (*FIL*), *CRABS CLAW* (*CRC*), *INNER NO OUTER* (*INO*), *YABBY2* (*YAB2*), *YABBY3* (*YAB3*), and *YABBY5* (*YAB5*) [7]. *FIL*, *YAB2*, *YAB3*, and *YAB5* are believed to play a role in the formation of vegetative and floral organs. The manifestation of *INO* and *CRC* is confined exclusively to the reproductive organs [8,9,10]. Various MADS proteins trigger *CRC*, which has a function in controlling the growth of nectaries and carpels. *INO* is distinctly present in the ovule primordium’s dorsal area, playing a role in creating and unevenly expanding the ovule’s external layer. *CRC* governs the growth of carpels and nectaries, showing expression trends akin to *AtCRC* in species including *Lagenaria siceraria*, *Chimonanthus praecox*, and *Ipomoea nil*. At present, the majority of YABBY gene family members in dicotyledonous plants play a role in determining the leaf antithetic cell properties and fostering leaf antithetic growth. However, the CRC-like genes of *Zea mays*, drooping leaf1 (drl1) and drooping leaf2 (drl2), showed no polar expression patterns in meristem [11]. Furthermore, research indicates the *YABBY* gene’s role in the biosynthetic process of plant secondary metabolism [12,13], For example, the *AtFIL* is important in controlling the synthesis of anthocyanin [13], while in *Artemisia annua*, *AaYABBY5* regulates the synthesis of artemisinin by amplifying the function of CYP71AV1 [12].

Known alternatively as *C. humilis*, the Rosaceae family member *C. humilis* is a Chinese-native wild fruit and medicinal plant [14,15]. The core component, known as “Yu Li ren” has been a longstanding ingredient in Chinese medicinal practices. The fruit is consumable fresh or can be transformed into dried fruit, vinegar, wine, juice, and jam. *C. humilis* and its products can serve as crucial raw materials in the manufacturing of healthcare items. *C. humilis* is abundant in carotenoids, flavonoids, phenols, and numerous other active compounds. Its potent antioxidant properties facilitate its highly promising application in the healthcare sector [16].

Recognizing the critical function of the *YABBY* gene in the growth of plants, the YABBY family has been the focus of much research on a genomic scale in diverse plant species, including 6 members of *A. thaliana* [7], 8 members of *Oryza sativa* [17], 17 members of *Glycine max* [4], 7 members of *Vitis vinifera* [18], and 9 members of *Ananas comosus* [19]. However, the YABBY family of *C. humilis* has not yet been documented. This study identified six *ChYABBY* genes of *C. humilis* and analyzed their evolutionary connections, chromosomal positioning, collinearity, and expression trends through bioinformatics-based approaches. Predictions were then made for the corresponding cis-regulatory components associated with hormones, the growth and development of plants, stress reactions, and light exposure. At the same time, the contents of total sugar and total flavone as well as antioxidant levels in fruits at four growth stages were measured. The comprehensive genomic examination of *C. humilis*’ YABBY gene family presented here yields fresh perspectives on the essential characteristics of Rosaceae fruits.

## 2. Materials and Methods

### 2.1. Data Sources

Full genomic sequencing and annotation data for *C. humilis* were sourced from the National Genome Data Center (https://ngdc.cncb.ac.cn/databases (accessed on 20 October 2024)) [20]. The registration number for genome assembly is GWHBCKI00000000. Information on the YABBY protein sequence (GCA_000005425.2) for *A. thaliana* was sourced from The Arabidopsis Information Resource (TAIR) database (https://www.arabidopsis.org/ (accessed on 20 October 2024)). The protein sequences for *V. vinifera*, *O. sativa*, *A. comosus*, and *Averrhoa carambola* were acquired from previous studies [2,21].

### 2.2. Description of Plant Material

The materials used in this experiment were the ‘Habo No.1’ variety of *C. humilis*. The samples were collected from an experimental orchard located in Harbin City, Heilongjiang Province, China. The tested plants were 5-year-old healthy trees with a planting row spacing of 0.5 m × 1.5 m. All the plants were grown in the open air under natural conditions. The average temperature during the growing season (from June to September) was 21.1 °C, and the total precipitation was approximately 260 mm, with most of the rainfall concentrated in July. The orchard adopted conventional water and fertilizer management. Once a year in the autumn, a well-decomposed organic fertilizer (about 15 kg/plant) was applied, and a compound fertilizer was applied during the fruit expansion period. Irrigation mainly relied on natural precipitation, and supplementary irrigation was carried out only during consecutive drought periods. The fruits were harvested at 4 development stages (YF: green fruits picked on 30 July 2023; GF: green fruits picked on 9 August 2023; SRF: red fruits picked on 22 August 2023; RF: red fruits picked on 1 September 2023), preserved at −80 °C, and subjected to RNA extraction.

### 2.3. Identification and Physicochemical Properties of the YABBY Gene in C. humilis

TBtools (V1.098) was employed to perform a local BLASTP search, referencing the YABBY protein sequence of *A. thaliana* [22]. Screening was conducted on the potential genes belonging to the YABBY gene family of *C. humilis*. We then acquired a pfam seed model (PF04690) from the Proteins Family Database (http://pfam.xfam.org/ (accessed on 20 October 2024)) and conducted a search in the domain using HMMER3.02 (http://hmmer.org (accessed on 20 October 2024)), maintaining standard settings [23]. The HMM (hidden Markov model) and BLASTP results were subsequently evaluated. To validate the dependability of the selected sequence, we employed the Conserved Domain Database (CDD) (https://www.ncbi.nlm.nih.gov/ (accessed on 20 October 2024)) to analyze the structural domain, guaranteeing the inclusion of all potential *ChYABBY* genes within the YABBY domain framework [24]. Following the manual elimination of repeated sequences, we analyzed the amino acid length, molecular weight, instability index, and isoelectric point of the ChYABBY protein using ExPasy (https://www.expasy.org/ (accessed on 20 October 2024)) [2,5]. Homologous modeling is extensively employed in the development of protein models [25]. Here, analogous protein configurations were employed as homology templates to determine the tertiary structure of the YABBY protein. Furthermore, the structures of six YABBY proteins were estimated using the SWISS-MODEL (https://www.swissmodel.expasy.org (accessed on 20 October 2024)) [19].

### 2.4. Phylogenetic Analysis

We examined the evolutionary lineage of the YABBY gene family in *C. humilis*, *V. vinifera*, *O. sativa*, *A. comosus*, and *A. carambola* by aligning multiple sequences of the YABBY proteins using MEGA11 [26]. For this analysis, we adopted a neighborhood linkage (NJ) technique to construct a phylogenetic tree comprising 1000 bootstrap copies [21,27]. Other parameters are default. The evolutionary tree was visualized using the online tool Evolview.

### 2.5. Gene Structure and Conserved Motif Analysis

Based on the GFF (general feature format) annotation files from the *C. humilis* genome database, TBtools was adopted to examine the exon–intron configuration of the *ChYABBY* genes. The online tool MEME (https://web.mit.edu/meme/current/share/doc/meme.html (accessed on 21 October 2024)) was employed for the discovery of preserved sequence patterns in the *YABBY* gene, setting the motif count at a maximum of 10 and other variables as default [28]. The motifs were visualized with TBtools [29].

### 2.6. Promoter Analysis

The DNA segment 2000 base pairs before the *YABBY* gene’s start codon (ATG) was isolated from the *C. humilis* genome using TBtools. PlantCARE (https://bioinformatics.psb.ugent.be/webtools/plantcare/html/ (accessed on 23 October 2024)) was then used to analyze the promoter region to forecast potential cis-acting elements, which were then visualized with TBtools [30].

### 2.7. Chromosome Localization and Collinearity Analysis

Gene duplication occurrences facilitate the generation of novel genes and their functions. TBtools was used for the intraspecific homology analysis of the tandem and segmental duplications of the *YABBY* gene in *C. humilis*. TBtools depicted the chromosomal positioning of the *ChYABBY* genes through the annotated GFF file of the *C. humilis* genome, and the Circos function in TBtools forecasted the genome’s collinearity. To determine the cross-species collinear links among *C. humilis* and *A. thaliana*, *M. pumila*, *V. vinifera*, and *O. sativa*, the genome sequence and GFF files of these species were examined using the one-step MCScanX function in TBtools. The Multiple Synteny Plot function in TBtools was then utilized for visual representation [22].

### 2.8. Gene Expression Analysis

The expression of the *ChYABBY* genes was examined through uniform transcriptome data sourced from earlier research [20,31]. The NCBI project login numbers for the original data are PRJNA636897, PRJCA005423. Custer analysis was then employed to examine the presence of *ChYABBYs* across various regions of *C. humilis* and throughout distinct growth phases (Young fruit (YF), 30 days after flowering; green fruit (GF), 42 days after flowering; slightly red fruit (SRF), 57 days after flowering; and red fruit (RF), 70 days after flowering) [31], and used log2 transformation (FPKM + 1) value to generate specific expression profile heat maps.

### 2.9. Real-Time Quantitative Analysis

Quantitative reverse transcription polymerase chain reaction (qPCR) was used to comprehensively examine the YABBY gene family expression in *C. humilis*. Initially, the extraction of total RNA from the fruit pulp was performed using the SPAREasy New Plant RNA Rapid Extraction Kit (AC0305) (Shandong Sparkjade Biotechnology Co., Ltd., Jinan, China). Reverse transcription was then conducted using a SureScriptTM first-strand cDNA synthesis kit (GeneCopoeia, Rockville, MD, USA). The resultant cDNA concentration was measured using a nanodrop spectrophotometer. The Primer3 online tool was subsequently used to create primers (Table S1), maintaining the Tm value between 55 °C and 60 °C. The *C. humilis* at various growth phases were identified using BlazeTaqTMSYBR^®^ GreenqPCRMix2.0, a real-time fluorescence quantitative PCR detection agent. The procedure used for qPCR is described as follows: 94 °C for 30 s; 45 sequences of 94 °C for 12 s, 58 °C for 30 s, 72 °C for 45 s; followed by heating for 1 s at 79 °C to aid in plate analysis. Following the final PCR phase, the temperature was raised from 55 °C to 99 °C at a rate of 0.5 °C s^−1^ to create a melting curve for the specimen. With the Actin gene serving as the internal benchmark, every reaction was conducted three times. The levels of expression were determined using the 2^−ΔΔCt^ technique [32].

### 2.10. Analysis of Potential Protein Interaction

The protein interaction network was constructed using STRING 11.0 (https://string-db.org/cgi/input.pl (accessed on 24 October 2024)). The network edges were set to confidence level, with parameters configured at medium confidence (0.400), and the display limited to no more than 10 interacting factors.

### 2.11. Determination of Total Sugar and Total Flavone Content

Follow the instructions of total sugar content kit and total flavone kit (Suzhou GrIS Biotech Co., Ltd., Suzhou, China). For the analysis of total sugars and flavonoids, fresh tissue samples (from Section 2.2) were extracted separately and were not the same extracts used for gene expression analysis.

### 2.12. Determination of Antioxidant Capacity

Follow the instructions of SOD, CAT, POD enzyme activity kit and MDA content determination kit (Suzhou Gris Biotechnology Co., Ltd.). The antioxidant capacity was determined using extracts prepared from fresh tissue samples (from Section 2.2) that were processed independently of the samples used for RNA extraction.

### 2.13. Statistical Analysis

Each group was repeated three times biologically. All results were expressed as mean ± standard error (SE). First, perform variance analysis on data such as gene expression levels, total sugar and total flavonoid contents, and antioxidant indicators. Then, conduct post hoc pairwise comparisons through the Bonferroni correction method to control for multiple comparisons. Finally, determine the differences at different growth stages. All statistical analyses of the data were conducted using SPSS 17.0, and all data were processed by GraphPad Prism 10.0. If *p* < 0.05, the difference was considered significant.

## 3. Results

### 3.1. Identification of the ChYABBY Gene and Its Physical and Chemical Properties

The AtYABBY protein sequence was obtained via the TAIR website. A search of the *C. humilis* genome was conducted using the YABBY sequence from *A. thaliana*. From the *C. humilis* genome, six members of the ChYABBY gene family were determined, and their protein sequences were integrated into the Pfam and NCBI CDD online tools to verify the conserved domains of YABBY. Based on their chromosomal locations, the genes are denoted as *ChYABBY1*–*ChYABBY6*. The proteins produced by the six *ChYABBYs* vary in length, spanning from 181 to 232 amino acids (Table S2). Each member of the ChYABBY family possesses a unique protein molecular weight. ChYABBY1 is the smallest protein, with a molecular mass of 19589.23 kDa, while ChYABBY4 is the longest protein, with a molecular mass of 25763.16 kDa. A significant variation is observed in the theoretical pI of the ChYABBY protein sequences, with values ranging from 5.41 (ChYABBY2) to 8.81 (ChYABBY3).

### 3.2. Structural Analysis of ChYABBY Protein and ChYABBY Gene

**Figure 1:**
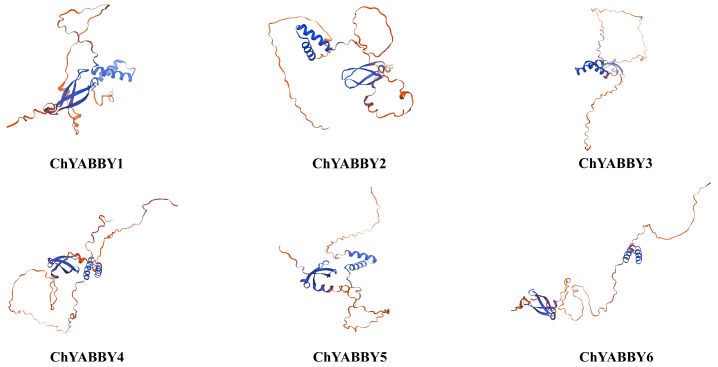
Predicted tertiary structures of the six ChYABBY proteins. The structures shown are in silico homology models generated using the SWISS-MODEL server. These models are hypothetical and have not been experimentally determined, serving primarily for the visualization of potential protein folds.

Figure 1 reveals the similar tertiary structures between ChYABBY3 and ChYABBY4 and between ChYABBY1 and ChYABBY5. The results show that the protein structures of ChYABBY members have similarities and differences, with differences observed even within the same subfamily. ChYABBY proteins typically comprise 4–6 motifs. Among them, motif 1 and motif 2 stand out as the most preserved—coding for YABBY and zinc finger domains—containing 50 and 48 amino acids, respectively. Moreover, distinct motifs specific to certain subgroups were identified, such as motif 4 and motif 10 in the YB3/FIL group. Generally, ChYABBY proteins within the same subfamily exhibit similar patterns of conserved motifs, with each protein displaying distinct conserved motifs across various subfamilies. Furthermore, the analysis of the *ChYABBY* gene structure indicates that the number of introns among the members of the YABBY family has not changed significantly (Figure S1). It is important to point out that the structures presented in Figure 1 are in silico predictions generated through homology modeling and have not been experimentally validated. Therefore, while useful for visualizing potential protein folds, these models are hypothetical, and any apparent structural similarities or differences should be simply interpreted as a possibility.

### 3.3. Phylogenetic Analysis

**Figure 2:**
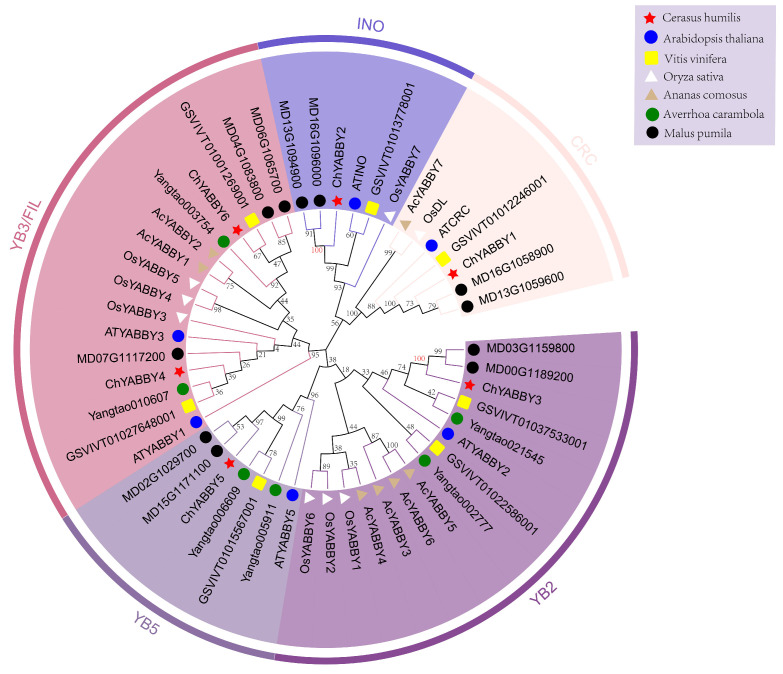
Phylogenetic analysis of the YABBY gene family in *C. humilis*, *A. thaliana*, *V. vinifera*, *O. sativa*, *A. comosus*, *A. carambola* and *M*. *pumila* using the NJ tree. The tree was constructed based on full-length protein sequences and included 51 YABBY members from six species.

To gain a deeper insight into the evolutionary links among *YABBY* genes, we developed a phylogenetic tree incorporating 51 *YABBY* genes from *C. humilis*, *A. thaliana*, *Pyrus bretschneideri*, *A. carambola*, *V. vinifera*, *O. sativa* and *M. pumila* (Figure 2). As an important model plant, *A. thaliana*’s *YABBY* genes have been extensively investigated to infer the function of *ChYABBY* genes. Previous phylogenetic research categorizes the *ChYABBY* genes into five subclades: FIL/YAB3 (2 members, *ChYABBY4* and *ChYABBY6*), YAB2 (1 member, *ChYABBY3*), YAB5 (1 member, *ChYABBY5*), INO (1 member, *ChYABBY2*), and CRC (1 member, *ChYABBY7*). The YAB5 lineage excludes *O. sativa* genes, aligning with earlier studies [23]. *ChYABBY3* forms a highly supported branch with *MD03G1159800* and *MD00G1189200* (bootstrap value > 95%), indicating that *ChYABBY3* shares a recent common ancestor with these two *M. pumila* homologous genes. Similarly, *ChYABBY2* forms a strongly supported branch with *MD13G1094900* and *MD16G1096000* (bootstrap value > 95%). Although the bootstrap values of some deep nodes defining the relationships among the major subfamilies are relatively low, this study focuses on classifying genes based on the high support rates of terminal branches.

### 3.4. Promoter Analysis

**Figure 3:**
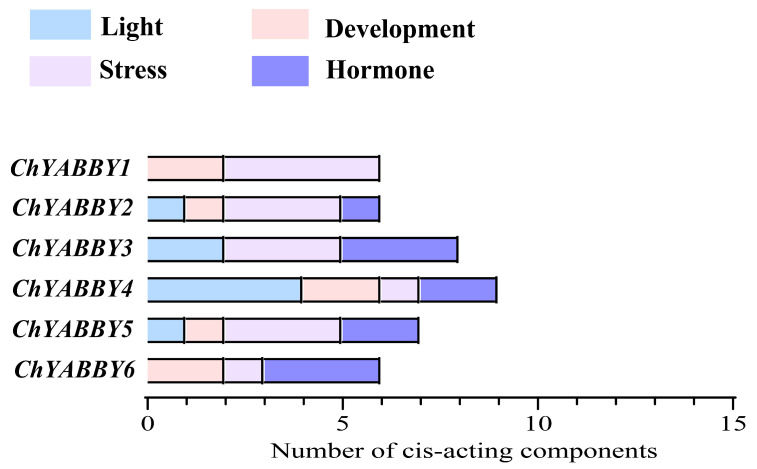
Summary of potential cis-acting elements predicted in *ChYABBY* gene promoters. The bar graph quantifies the number of putative regulatory motifs identified via an in silico search of the 2 kb upstream region of each gene. Motifs are categorized based on their potential function according to the PlantCARE database. These results are predictive and do not represent experimentally verified regulatory sites.

We examined the cis-acting components of the *ChYABBY* promoter sequence to understand how *ChYABBY* reacts to biotic and abiotic stressors. In Figure 3, 11 distinct cis-acting components were discovered within 6 *ChYABBYs.* Initially, our discovery encompassed several plant hormone response components, such as abscisic acid (ACGTG motif), salicylic acid (CCATCTTTTT motif), and gibberellin (TCTGTTG motif). In addition, we identified elements in the promoter that respond to stress, including those in drought (CAACTG motif), low-temperature (CCGAAA motif), anaerobic (AAAACCA motif), and hypoxic environments. A variety of photoresponsive components were observed to be extensively dispersed across the six *ChYABBYs*, with a notably high count of these elements. The results reveal that various *ChYABBYs* possess distinct cis-acting components. Within the same subgroup, the variety and quantity of cis-acting elements exhibit differences. This suggests that the *ChYABBY* genes may be involved in diverse physiological and biochemical processes, with the specific roles potentially varying across the growth and maturation of plants. It is crucial to recognize that the in silico pattern-matching search that identifies short, potential regulatory motifs, is predictive and does not constitute experimental evidence of gene function. The identification of these motifs is a starting point for generating hypotheses, but their biological roles in regulating gene expression in Cerasus humilis must be confirmed through experimental validation, such as promoter-reporter assays or ChIP-qPCR.

### 3.5. Genomic Distribution and Duplication of ChYABBY Genes

The six identified *ChYABBY* genes are distributed across five different chromosomes, with no evidence of local tandem duplications. An analysis of large-scale segmental duplications revealed only one paralogous pair (*ChYABBY4* and *ChYABBY6*), suggesting that gene duplication has not been a major driver of the YABBY family’s expansion in the Cerasus humilis lineage. A comparative synteny analysis confirmed a high degree of conserved genomic blocks between C. humilis and the closely related Malus pumila, which provides a framework for future studies (Figures S2 and S3 and Text S1). However, as a detailed gene-by-gene orthology analysis was beyond the scope of this initial study, these genomic comparisons cannot yet be translated into specific functional hypotheses for individual *ChYABBY* members.

### 3.6. Targeted Validation of ChYABBY Gene Expression by qRT-PCR

To investigate the potential role of *ChYABBY* genes during fruit development, we performed a targeted gene expression analysis. As an initial exploratory step to guide this analysis, we first re-analyzed publicly available transcriptome datasets to generate a preliminary expression heatmap. This step was used solely for hypothesis generation and to prioritize the subsequent experimental validation. Given the well-documented limitations of FPKM normalization and Z-score visualization for quantitative comparisons, this preliminary heatmap should be interpreted with caution and is available in the Supplementary Materials (Figure S4). To obtain robust and quantitative expression data, we then performed qRT-PCR on newly collected biological replicates from four key stages of fruit development. The results of this quantitative analysis are presented below.

### 3.7. Real-Time Quantitative Analysis

**Figure 4:**
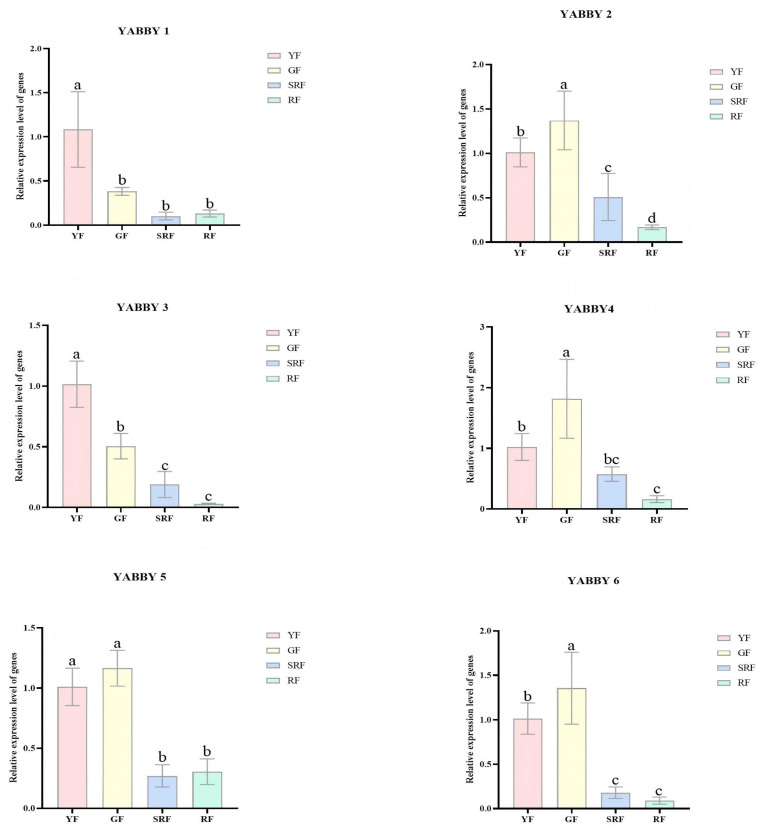
qRT-PCR analysis of the *ChYABBY* gene. YF: green fruits picked on 30 July 2023; GF: green fruits picked on 9 August 2023; SRF: red fruits picked on 22 August 2023; RF: red fruits picked on 1 September 2023. Different letters indicate a statistically significant difference between groups as determined by a one-way ANOVA followed by a Bonferroni post hoc test (*p* < 0.05).

**Figure 5:**
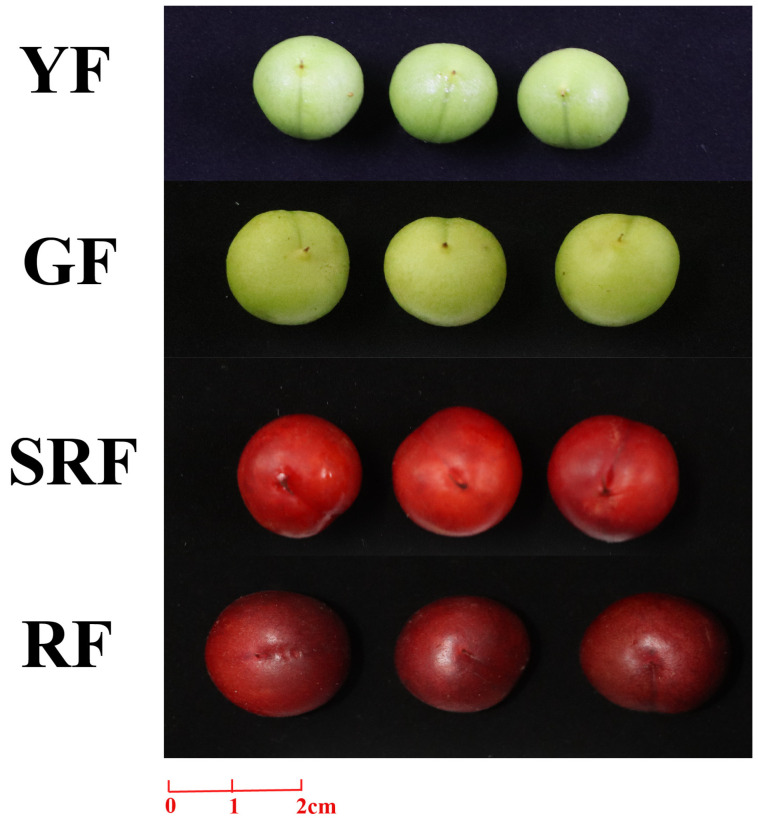
Fruit photos of four growth points of *C*. *humilis*.

The heat map in Figure 4 illustrates that the *ChYABBY* gene plays a role in the growth and development of *C. humilis*. To confirm this, qPCR was conducted on the *ChYABBY* genes at four distinct growth phases of the fruit in Figure 5. We found that *ChYABBY* genes were mainly expressed in *C. humilis*’s green fruits, among which *ChYABBY2*, *ChYABBY4*, *ChYABBY5* and *ChYABBY6* were mainly expressed in GF group, while the other two were mainly expressed in YF group.

### 3.8. Inferred Protein Interaction Network Based on Homology

**Figure 6:**
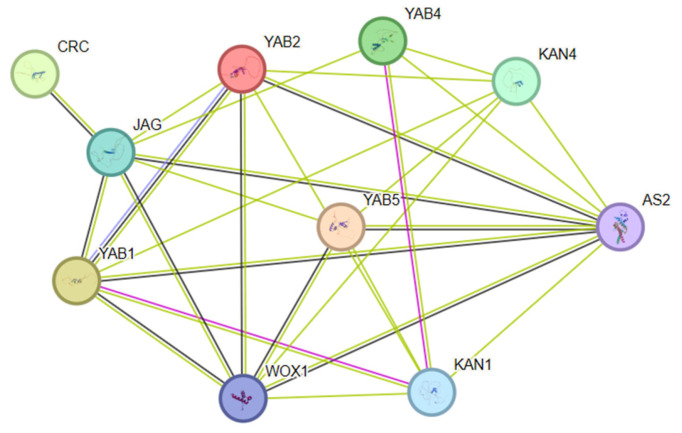
Inferred protein interaction network for ChYABBY proteins based on known interactions in model species. This network was generated using the STRING database. The lines connecting the proteins represent inferred functional associations that are transferred from experimentally validated interactions of homologous proteins (primarily from *Arabidopsis thaliana*). The different line types between nodes indicate the types of evidence for the association: Text mining (green), co-expression (black), experimentally determined (pink), and database annotated (blue).

To generate hypotheses about the potential functional context of the ChYABBY proteins, their sequences were submitted to the STRING database. It is critical to understand that the resulting network (Figure 6) does not represent experimentally verified or directly predicted interactions within Cerasus humilis. Instead, it is an inferred network based on transferring known, experimentally validated interactions from the corresponding homologous proteins in other species, predominantly Arabidopsis thaliana. Although Arabidopsis is phylogenetically distant from *C. humilis* and the number of annotated homologous proteins available for such comparisons is limited, this approach currently provides a framework for exploring putative, candidate functional associations to be experimentally tested in the future. For example, the predicted interaction between ChYABBY1 (a CRC homolog) and a JAG homolog is based on the well-documented physical interaction of these proteins in Arabidopsis. Therefore, this graphical representation should be interpreted not as a result, but as a set of specific, testable hypotheses for future experimental validation, such as yeast two-hybrid or co-immunoprecipitation assays.

### 3.9. Determination of Total Sugar and Total Flavone Content

**Figure 7:**
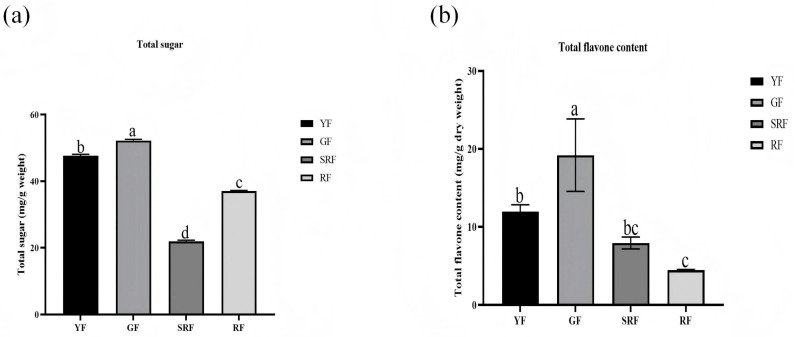
Determination of total sugar (**a**) and total flavone (**b**). Different letters indicate a statistically significant difference between groups as determined by a one-way ANOVA followed by a Bonferroni post hoc test (*p* < 0.05).

As can be seen from Figure 7, total sugar content in GF group is the highest, while SRF group is the lowest. Total flavonoids content in GF group was the highest, while RF group was the lowest. In addition, it can be seen from Figure 7 that the content of total sugar and total flavonoids in green fruits is significantly higher than that in red fruits, which is consistent with the expression changes in *YABBY* genes in the four fruits.

### 3.10. Determination of Antioxidant Capacity

**Figure 8:**
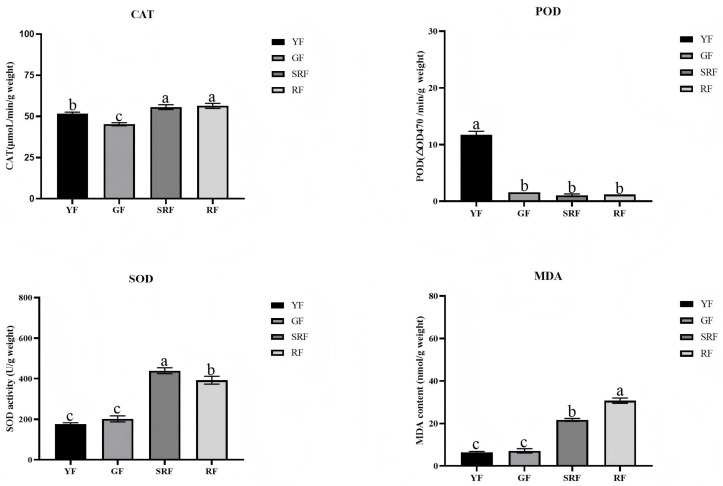
Determination of antioxidant capacity. Different letters indicate a statistically significant difference between groups as determined by a one-way ANOVA followed by a Bonferroni post hoc test (*p* < 0.05).

It can be seen from Figure 8 that different oxidation indexes have different change trends during fruit growth. Among them, SOD and CAT activities in green fruits are lower than those in red fruits, while POD activities are higher in early green fruits, which may be because different antioxidant enzymes play a major role in different periods of fruit growth. The content of MDA in red fruit is higher than that in green fruit, which may be due to the increase in damage degree caused by environmental influence along with fruit growth.

## 4. Discussion

The *YABBY* genes are crucial in the development of plant fruits. Reports indicate that *Solanum lycopersicum* contains nine *YABBY* genes. Moreover, *FASCIATED* (*SlYABBY2b*) is reported to primarily regulate pericenter formation during the flowering and fruit growth of *S. lycopersicum*. The *SlYABBY1a* gene, known for its high expression in the epidermis, is suggested to influence the growth of distal pericarp cells [33,34,35]. The *YABBY* gene has been identified in the fruits of some plants, such as *A. comosus* [19], *V. vinifera* [18], and *Fragaria* × *ananassa* [36]. The relatively small number of identified members may reflect the lack of expansion of this family during evolution. Given that the *YABBY* genes of *C. humilis* and *M. pumila* have a considerable number of subgroups and adjacent evolutionary paths, it is hypothesized that they have a close genetic connection. However, the majority of the branch relationships in this evolutionary tree remain unclear, and the deep connections among the gene lineages cannot be determined through statistical methods. The evolutionary differentiation process of gene structure implies the diversification of gene functions. For example, within *A. thaliana*, *CRC* plays a role in creating nectaries and carpels, whereas *INO* contributes to the formation of the integumen [7]. Similarly, the functions of *ChYABBY* may be diverse.

Analysis of cis-regulatory elements indicates *ChYABBYs* are characterized by numerous elements responsive to light, suggesting that light can influence the expression of *ChYABBY* genes. This agrees with studies on *LsaYABs* and *MdYABBYs* [2,5]. Previous literature has determined the importance of the *YABBY* gene in biological processes [2,4,37]. Our results suggest that the *ChYABBY* gene may have a function in reactions to abiotic stress factors. This twofold role implies that the *YABBY* gene could be important in regulating development and orchestrating reactions to environmental adversities. Furthermore, components that react to abscisic acid (ABA) and gibberellin acid (GA) frequently appear in members of the ChYABBY family. Abscisic acid plays a role in controlling the growth and maturation of plants, overcoming osmotic obstacles, and resisting negative environmental elements [38,39]. GAs are crucial in multiple facets of plant growth and maturation, encompassing stem development and blooming [40]. Furthermore, ABA and JA play a significant role in the development of fruits, encompassing the commencement of ripening, the overall ripening process, and fruit quality characteristics [41]. By enhancing the activity of genes involved in ethylene biosynthesis, they facilitate the maturation of various fruits, as evidenced in research on *Prunus salicina*, and *S. lycopersicum*, among others [3]. The abundance of ABA and GA response elements indicates that the *YABBY* gene may complexly regulate the growth and development of *C. humilis* through the ABA and GA signaling pathways [11]. The expression of *ChYABBY* genes in different tissues and growth stages further supports the hypothesis that these genes play a key role in growth regulation. While the analysis of cis-acting elements in promoter regions provides a valuable roadmap, these are in silico predictions. The actual regulatory impact of these elements needs to be confirmed experimentally, for example, through promoter-reporter assays.

The replication of genes, a frequent occurrence in evolution, happens as the genome of an organism obtains an additional copy of a pre-existing gene. Various processes, including the unequal crossover, retrotransposition, and duplication of the entire genome, can lead to this outcome. The duplication of genes is vital in evolutionary processes, fostering chances for functional variation and innovation. Genes that recur may develop novel roles via diverse processes such as subfunctionalization and neofunctionalization, thus enhancing the complexity and variety within organisms. Within the ChYABBY gene family, a singular segmental duplication occurrence was predominantly noted, with an absence of tandem duplication events. This discovery aligns with the outcomes of the *YABBY* homology study conducted on *O. sativa*, *Solanum tuberosum*, *Cicer arietinum*, and *Olea europaea*, among other plants [42]. Moreover, the primary catalyst for the expansion of a species’ genome is gene duplication, and the reduced occurrence of *ChYABBYs* duplications also accounts for the limited gene family members. Variations in the gene family across diverse eukaryotic species could stem from evolutionary mechanisms. To further investigate deeper into the development of *YABBY* members, an analysis of interspecific collinearity was conducted among *C. humilis*, *A. thaliana*, *O. sativa*, *M. pumila*, and *V. vinifera* to explore their interrelations. The results indicate that the *YABBY* gene of *C. humilis* exhibits the highest count of collinearities with *M. pumila*, a member of the Rosaceae family. Notably, a single gene in *C. humilis* exhibits collinearity with several genes in *O. sativa* and *M. pumila*. This suggests that these homologous *ChYABBY* genes are conserved and may have existed before their common ancestor diverged.

Previous studies have shown that *YABBY* plays a significant role in the growth and development of plants and organs [43,44]. This study systematically analyzed the expression patterns of six *ChYABBY* genes in various tissues and four key fruit development stages (YF, GF, SRF, RF) through integration of transcriptome analysis (Section 2.7) and qPCR verification (Section 2.8), providing important evidence for revealing their potential functions in the specific organ development of *C. humilis*, especially in the process of flower and fruit development. The transcriptome heatmap analysis (Figure S4b) clearly demonstrates that the expression of the *ChYABBY* gene exhibits a high degree of tissue specificity. The six *ChYABBY* genes are hardly expressed in roots and stems, with only *ChYABBY5* showing weak expression in leaves. In flowers and fruits, they exhibit significant and varying degrees of high expression. This strongly suggests that the members of the *ChYABBY* gene family mainly participate in the development of reproductive organs (flowers and fruits) of *C. humilis*, rather than in vegetative growth. It is worth noting that this is consistent with the reports that certain *YABBY* members in Arabidopsis (such as CRC and INO) mainly function in reproductive growth, indicating functional conservation [6]. In Figure S4b, *ChYABBY1* and *ChYABBY2* showed specific expression in flowers and fruits. However, Figure S4a revealed that these genes were not detected in the fruits at the four developmental stages. *ChYABBY1* and *ChYABBY2* might be expressed only in other stages of fruit development not covered by Figure S4a (such as earlier or later stages). This suggests that *ChYABBY* may regulate a very specific and brief window period in fruit development, and the specific expression dynamics and regulatory mechanisms of *ChYABBY* need to be further clarified in future studies (such as more frequent time point sampling). Additionally, unlike the fruit development data (Figure S4a), which was derived from a uniformly processed and replicated dataset, the cross-tissue comparison (Figure S4b) was collected from different public sources and may differ in terms of experimental design and sequencing depth, and it lacks biological replication. Our exploratory analysis of public RNA-seq data was designed to identify broad expression patterns rather than perform a rigorous statistical test of differential expression. A future, dedicated transcriptome study using count-based methods like DESeq2 or edgeR would be required to definitively identify statistically significant expression changes.

The use of FPKM values and Z-score normalization was adopted for initial exploratory visualization and cross-tissue comparison; however, FPKM is no longer the preferred metric for quantitative cross-sample comparisons due to its inability to account for transcript length biases across genes and samples. Moreover, while Z-scores help illustrate relative expression patterns, they may mask differences in absolute expression levels. This approach may affect the reliability of direct quantitative comparisons across tissues. These considerations highlight the importance of our subsequent q-PCR validation, which provides more robust and biologically validated support for the expression patterns described. The results showed that the *ChYABBY* genes were mainly expressed in green fruits, which was consistent with the expression trend of the transcriptome data. This indicates that the *ChYABBY* genes play an important role in regulating fruit development. The use of a single cultivar, ‘Habo No.1’, provides a crucial first look, but the conservation of these gene expression patterns and their physiological correlations should be investigated across a broader range of Cerasus humilis germplasm. It is well-established that genotype-by-environment interactions can significantly impact fruit ripening. Therefore, the trends observed here should be considered a baseline that requires validation under different horticultural and climatic conditions.

Based on the predicted results of protein interactions, this study speculates that the YABBY protein of *C. humilis* may regulate fruit development through conserved interaction modules. In *Arabidopsis*, CRC and JAG interact to promote cell proliferation and morphogenesis in the carpel and valve tissues. Similarly, this study predicts that the interaction between *ChCRC* and ChJAG may promote the proliferation and expansion of *C. humilis* young fruit wall cells, laying the foundation for early fruit growth. YABBY protein and AS2 synergistically inhibit *KNOX* gene expression in *Arabidopsis* to maintain deterministic growth of leaves and flower organs. Consistent with this, ChYAB1/2/5 may form a complex with ChAS2, which maintains the deterministic growth of fruit peels by inhibiting genes such as ChKNOX, promoting cell differentiation and enlargement. In addition, the YABBY-KAN module in *Arabidopsis* is a key regulatory unit for establishing dorsal ventral polarity in lateral organs. Similarly, the interaction between ChYAB1/4 and ChKAN1 may synergistically regulate the radial polarity of the ovary wall, promoting the development of the outer and middle pericarp and allowing the inner pericarp to harden, thereby ensuring the formation of typical drupe structures. These interactive networks may work synergistically during the fruit development of *C. humilis*, and their functional mechanisms may be highly similar to the conserved regulatory modules in *Arabidopsis.* Furthermore, the protein–protein interaction network presented in Figure 6 is based on predictions from homologous proteins in model species and does not represent experimentally verified interactions in Cerasus humilis.

In the process of fruit growth and development, sugar is the direct use of carbon nutrition. It is speculated that due to the vigorous growth of green fruit, the total sugar content is higher than that of red fruit, and the total sugar content is positively correlated with the expression of *YABBY* gene, which may be that sugars provide energy in the life activities regulated by *YABBY* gene. It has been reported that *YABBY* gene is a positive regulator of flavonoid biosynthesis in *Artemisia caruifolia*. The expression of flavonoid biosynthesis gene and total flavonoid content in *A. caruifolia* strains overexpressed by *YABBY5* gene continued to increase, and the expression of flavonoid biosynthesis gene and total flavonoid content significantly decreased after *YABBY5* antisense inhibition [45]. In addition, *YABBY1* can promote the synthesis of flavonoids in tobacco [46]. In this study, the expression of *YABBY* gene was consistent with the content of total flavonoids, suggesting that *ChYABBY* gene might promote the synthesis of flavonoids in *C. humilis*.

The activity of antioxidant enzymes such as SOD, POD and CAT is an important index to evaluate the antioxidant level of plants. SOD is the primary substance to remove free radicals in fruit, and can catalyze the disproportionation reaction of O_2_^−^ to produce O_2_ and H_2_O_2_. POD and CAT can remove excess reactive oxygen species and avoid oxidative damage. In this study, the activity of the three antioxidant enzymes showed different changes during fruit growth, indicating that different enzymes play the main antioxidant role at different developmental stages [47]. In green fruits with high *YABBY* gene expression, we observed higher activities of CAT and POD. We speculate that this phenomenon might be linked to the role of the *YABBY* gene in flavonoid synthesis. Flavonoids are known to support antioxidant defense by, for example, enhancing the activities of enzymes like catalase and glutathione peroxidase, while inhibiting ROS-producing enzymes [48]. Therefore, it is plausible that the *YABBY* gene could contribute to improved fruit antioxidant capacity via the flavonoid-mediated pathway. However, further research is necessary to validate this proposed mechanism. A central limitation remains the absence of direct functional genetics. While our data establish a compelling correlation, causal relationships can only be confirmed through the functional disruption or overexpression of key *ChYABBY* candidates. Therefore, the proposed regulatory roles for specific *ChYABBY* genes in flavonoid or sugar accumulation should be viewed as well-supported hypotheses that now require rigorous experimental testing.

## 5. Conclusions

We performed the first systematic genome-wide bioinformatics analysis of *ChYABBYs* in *C. humilis*. Six *ChYABBY* genes were identified and categorized into five subfamilies. Genes in the same subclade share similar conserved motifs and structures, yet each has unique sequence characteristics outside of conserved amino acid domains. The chromosome distribution appears to be independent of phylogenetic relationships. Containing multiple types of promoters implies functional diversity. Collinearity analysis showed that there is only one segment duplication in the genome of *C. humilis*, which also explains its small number of gene family members. *C. humilis* exhibits the highest number of collinearities with *M. pumila*. This may be because both species belong to the Rosaceae family. Although our collinearity analysis provides a basic evolutionary framework, the lack of individual orthologous analysis currently prevents us from translating these genomic comparisons into specific functional hypotheses for individual *ChYABBY* members. Establishing precise orthologous relationships with model species remains a key goal for future research. Our initial expression analysis was based on a re-analysis of publicly available RNA-seq datasets. We acknowledge that the use of FPKM for normalization and Z-scores for visualization is primarily suited for identifying expression patterns rather than making precise quantitative comparisons. Recognizing these limitations, we performed targeted qRT-PCR analyses on newly collected biological replicates to validate the key trends observed during fruit development, thereby providing a more robust quantitative foundation for our main conclusion. The results showed that the *ChYABBY* genes were mainly expressed in green fruits. This indicates that the *ChYABBY* genes play an important role in regulating fruit development. The results of this study aid in understanding the functional characteristics of the YABBY gene family of *C. humilis*, and also provide comprehensive information and new insights into the functions of *YABBY* genes in plants. Furthermore, as the experimental data for this study were collected from a single growing season and specific orchard conditions, the observed trends in gene expression and fruit physiology should be considered preliminary. Future studies across multiple years and diverse environments will be necessary to confirm the quality of these findings and the magnitude of environmental influences.

## Data Availability

Data will be made available on request.

## Supplementary Materials

The following supporting information can be downloaded at https://www.mdpi.com/article/10.3390/biology14111511/s1, Figure S1: Analysis of Gene Structure and Protein Motifs Figure S2: Intraspecific and Interspecific Correlation Analysis; Figure S3: Chromosome Localization Analysis; Figure S4: Gene Expression Analysis; Table S1: qPCR Primers; Table S2: Analysis of Protein Physicochemical Properties.
