# Supplementary material for: Identification of the YABBY Gene Family in Cerasus humilis and Analysis of Expression Patterns During Different Growth Stages
Source: Biology (Basel). 2025 Oct 28;14(11):1511. doi: 10.3390/biology14111511 (PMC12649974; doi:10.3390/biology14111511)

ChYABBY3

Sort	Coverage	GMQE	QSQE	Identity	Method	Oligo State	Ligands
✓	A0A6P5TP43.1.A Axial regulator YABBY 2 isoform X1<br>*AlphaFold DB model of A0A6P5TP43_PRUAV (gene: LOC110769164, organism: Prunus avium (Cherry) (Cerasus avium))*						
		0.59	-	97.92	AlphaFold v2	monomer ✓	None

Ramachandran Plots

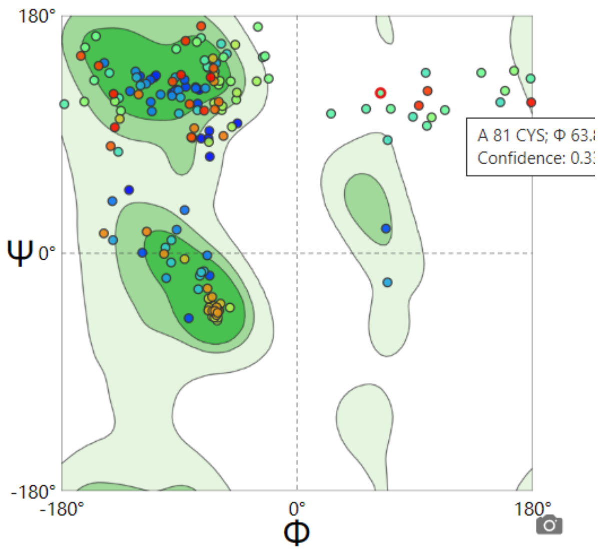

ChYABBY5

Sort	Coverage	GMQE	QSQE	Identity	Method	Oligo State	Ligands
✓	C6SYG8.1.A Uncharacterized protein<br>*AlphaFold DB model of C6SYG8_SOYBN (gene: C6SYG8_SOYBN, organism: Glycine max (Soybean) (Glycine hispida))*						
		0.66	-	86.49	AlphaFold v2	monomer ✓	None

Ramachandran Plots

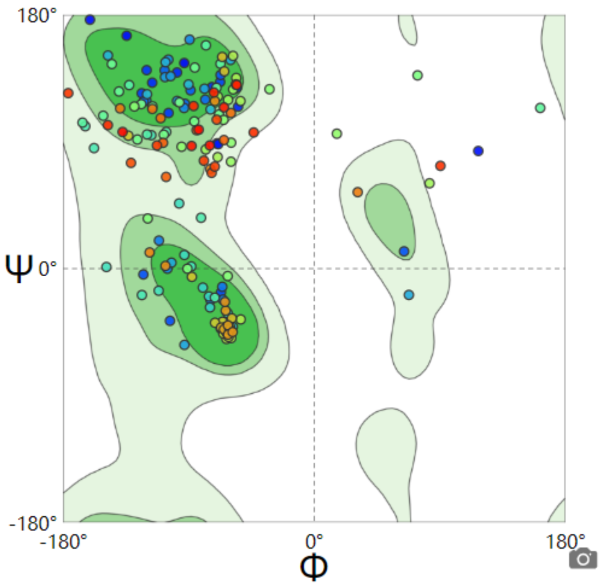

ChYABBY4

Templates | Quaternary Structure | Sequence Similarity | Alignment | More

Sort	Coverage	GMQE	QSQE	Identity	Method	Oligo State	Ligands
I1J7S5.1.A Axial regulator YABBY 1<br>*AlphaFold DB model of I1J7S5_SOYBN (gene: LOC100781267, organism: Glycine max (Soybean) (Glycine hispida))*							
		0.59	-	82.16	AlphaFold v2	monomer ✓	None

Ramachandran Plots

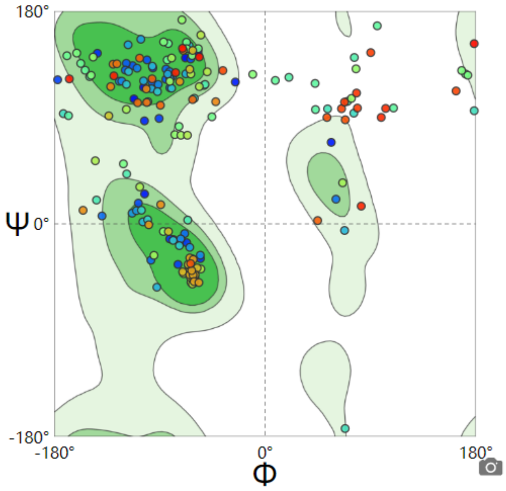

ChYABBY1

Sort	Coverage	GMQE	QSQE	Identity	Method	Oligo State	Ligands
K7K269.1.A Uncharacterized protein<br>*AlphaFold DB model of K7K269_SOYBN (gene: LOC100795791, organism: Glycine max (Soybean) (Glycine hispida))*							
		0.62	-	77.71	AlphaFold v2	monomer ✓	None

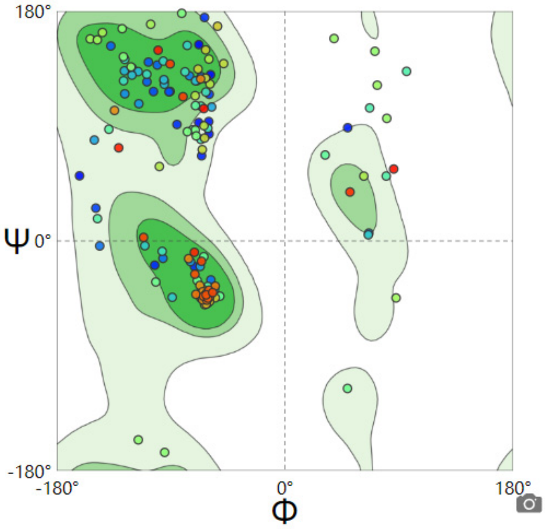

ChYABBY2

Sort	Coverage	GMQE	QSQE	Identity	Method	Oligo State	Ligands
✓	A0A834TWP3.1.A Axial regulator YABBY 4<br>*AlphaFold DB model of A0A834TWP3_9FABA (gene: A0A834TWP3_9FABA, organism: Senna tora)*						
		0.59	-	71.03	AlphaFold v2	monomer ✓	None

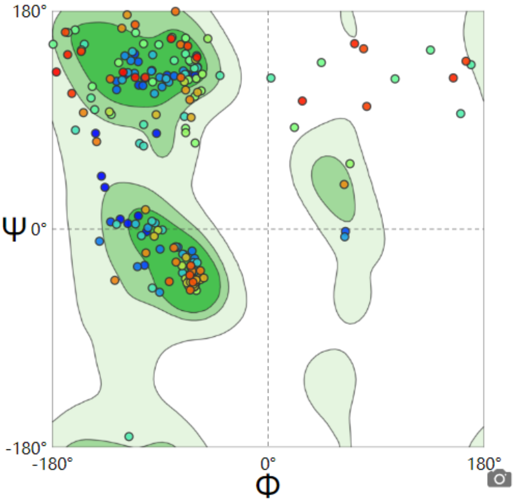

ChYABBY6

Sort	Coverage	GMQE	QSQE	Identity	Method	Oligo State	Ligands
✓	I1K0L1.1.A Uncharacterized protein<br>*AlphaFold DB model of I1K0L1_SOYBN (gene: LOC100786175, organism: Glycine max (Soybean) (Glycine hispida))*						
		0.62	-	89.05	AlphaFold v2	monomer ✓	None

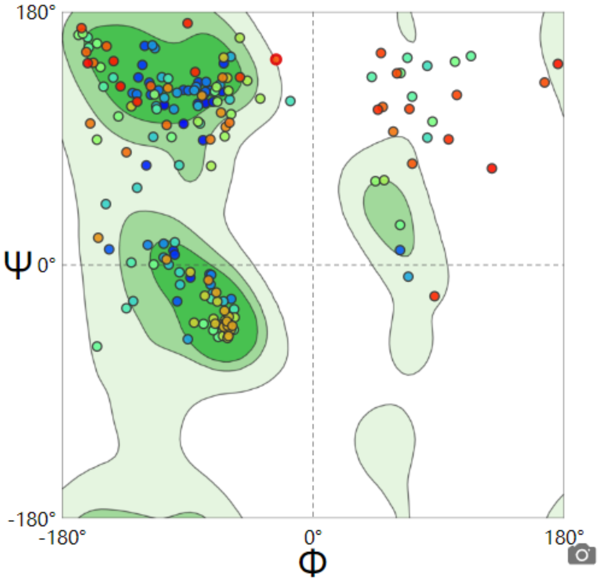

Supplement: Supplementary file 1 [file biology-14-01511-s001.zip › 3D protein structure modeling.pdf]
