# Supplementary material for: Identification of the YABBY Gene Family in Cerasus humilis and Analysis of Expression Patterns During Different Growth Stages
Source: Biology (Basel). 2025 Oct 28;14(11):1511. doi: 10.3390/biology14111511 (PMC12649974; doi:10.3390/biology14111511)
Supplement: Supplementary file 1 [file biology-14-01511-s001.zip › Supplementary Text.pdf]

Gene mapping analysis revealed an irregular distribution of the six ChYABBY genes across the genome chromosomes. ChYABBY1 and ChYABBY2 reside on chromosome 1; ChYABBY3 on chromosome 2; ChYABBY4 on chromosome 3; ChYABBY5 is situated on chromosome 4; and ChYABBY6 resides on chromosome 8. However, the ChYABBY gene is absent from chromosomes 5, 6, and 7 (Supplementary Figure 3). Additionally, There is no tandem duplication in the ChYABBY gene family, with just a single pair of segmentally duplicated genes present (ChYABBY4 and ChYABBY6).

In order to trace the evolutionary trajectory of the ChYABBY gene family, we analyzed the homology relationships between C. humilis and four reference species (A. thaliana, M. pumila, V. vinifera, and O. sativa) (Figure 4). The number of homologous gene pairs between C. humilis and A. thaliana (3 pairs) was less than that between C. humilis and V. vinifera (5 pairs), O. sativa (6 pairs), and M. pumila (9 pairs). This homology pattern of the YABBY genes - especially the maximum homology with M. pumila - is consistent with the phylogenetic evidence. In the evolutionary tree, ChYABBY2 and ChYABBY3 form a branch together with their M. pumila homologous genes, indicating that the evolution of these specific lineages is conserved. Overall, the high homology of YABBY genes between C. humilis and M. pumila and their close phylogenetic relationship are likely due to their common Rosaceae ancestor and recent divergence. This study provides important clues and references for understanding the functional differentiation of the YABBY gene family in economic Rosaceae crops.
