# Supplementary figures and images for: Identification of the YABBY Gene Family in Cerasus humilis and Analysis of Expression Patterns During Different Growth Stages
Source: Biology (Basel). 2025 Oct 28;14(11):1511. doi: 10.3390/biology14111511 (PMC12649974; doi:10.3390/biology14111511)

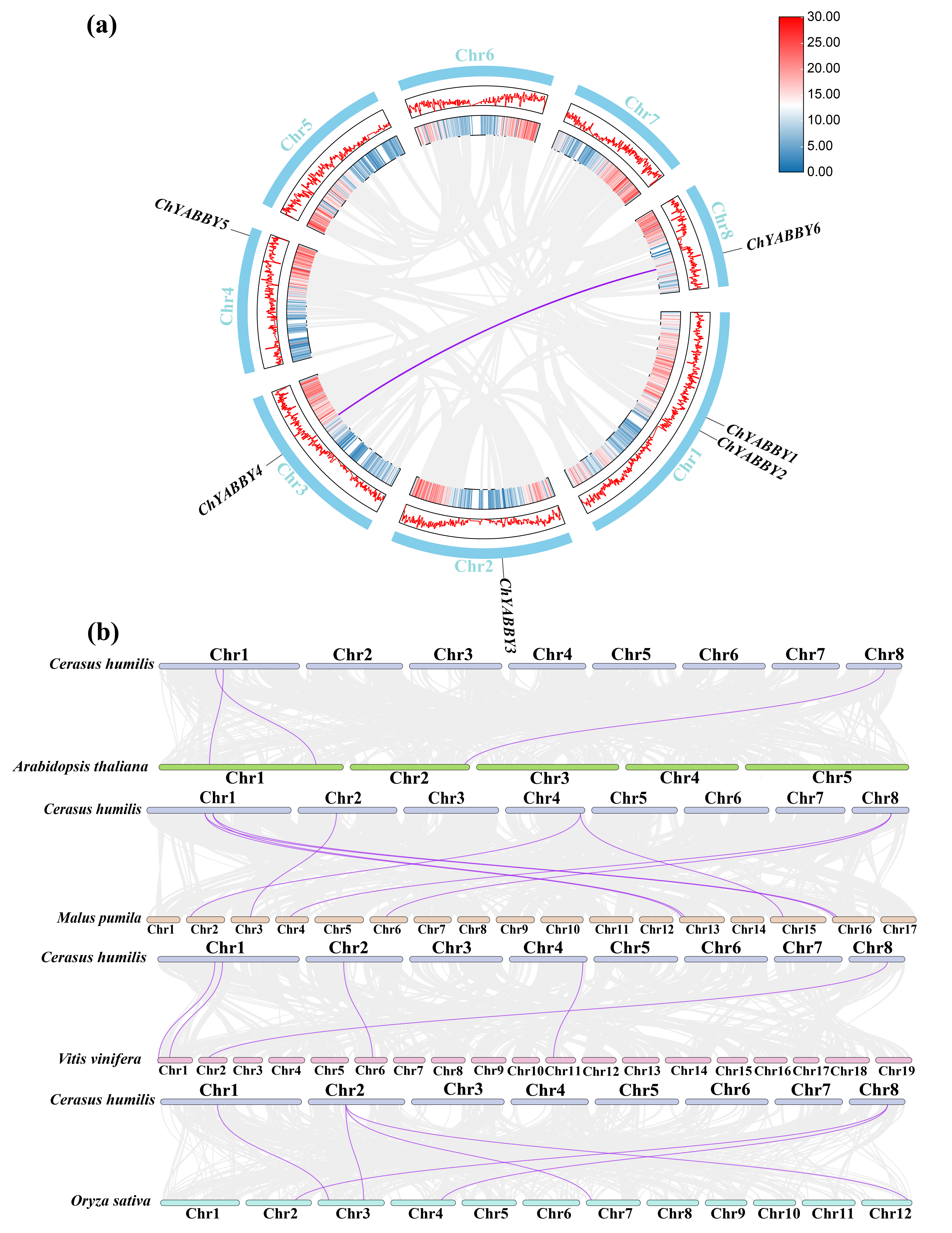

Supplement: Supplementary file 1 [file biology-14-01511-s001.zip › Figure S2.jpg]

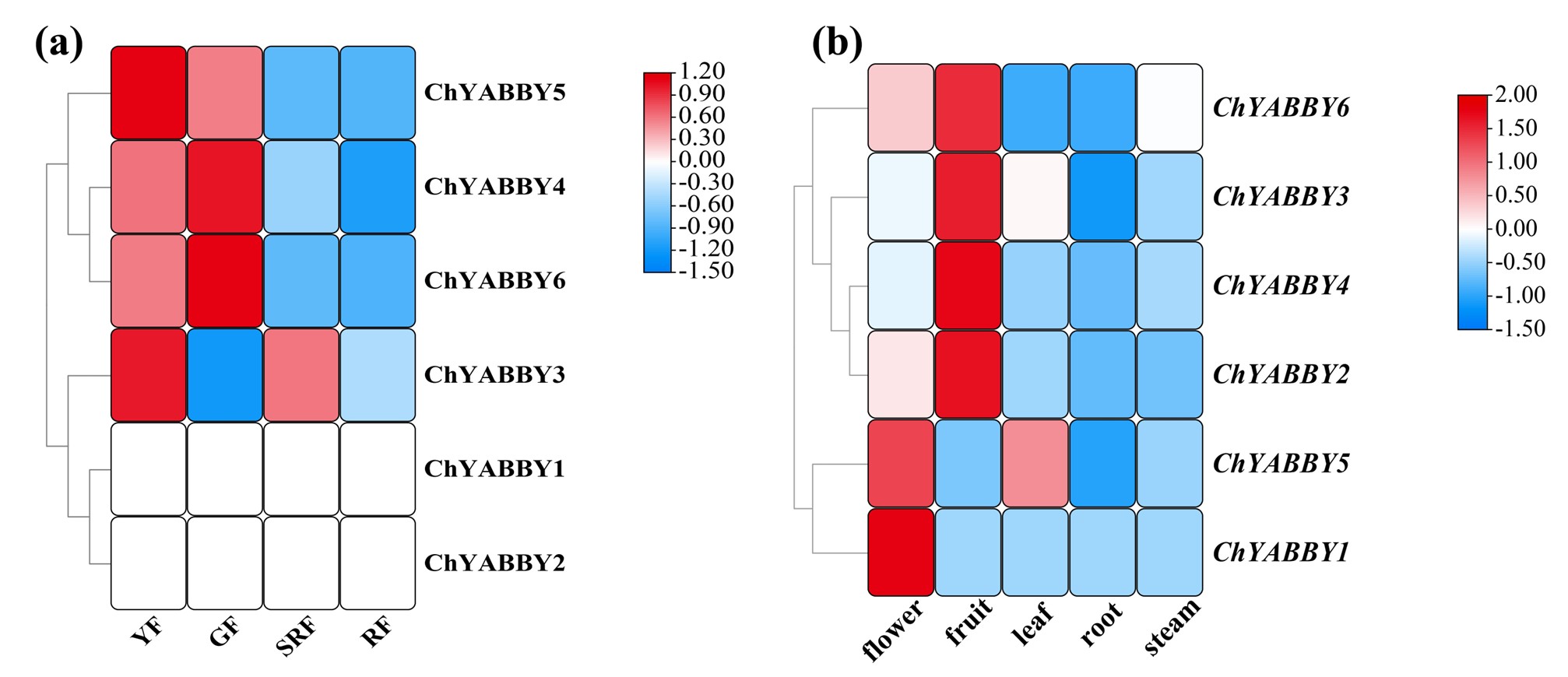

Supplement: Supplementary file 1 [file biology-14-01511-s001.zip › Figure S4.jpg]
